# Weakly Coordinated Solvent Network‐Induced Ternary Water‐Deficient Eutectic Electrolytes for Low‐Polarization Zn Anodes

**DOI:** 10.1002/advs.77566

**Published:** 2026-09-06

**Authors:** Quancai Li, Jing Liang, Jiayu Wang, Qian Wang, Hehe Ren, Ziyi Gong, Vellaisamy A. L. Roy, Wei Wu

**Affiliations:** ^1^ Laboratory of Printable Functional Materials and Printed Electronics School of Physics and Technology Renmin Hospital of Wuhan University Wuhan University Wuhan People's Republic of China; ^2^ School of Science and Technology Hong Kong Metropolitan University Ho Man Tin Hong Kong People's Republic of China; ^3^ Department of Clinical Laboratory Institute of Translational Medicine Renmin Hospital of Wuhan University Wuhan People's Republic of China

**Keywords:** eutectic electrolyte, solid‐electrolyte interphase, solvation structure, zin‐ion batteries

## Abstract

Eutectic electrolytes are attractive for zinc‐based energy storage owing to their green chemistry attributes and intrinsic stability; however, their practical deployment is limited by severe polarization stemming from sluggish ion transport and strong solvation. Here, we propose a ternary water‐deficient eutectic electrolyte without water solvent whose polarization approaches that of aqueous systems by engineering a weakly coordinated solvent network via solvation‐structure restructuring. A strongly coordinated solvent network enables salt dissolution, whereas the introduction of a weakly coordinating co‐solvent disrupts the constrained coordination environment and drives a transition to a dynamic, weakly coordinated solvation structure. This restructuring enhances anion participation while suppressing ion bridging, yielding abundant yet size‐confined aggregates, thereby accelerating ion‐transport kinetics and lowering desolvation barriers. Furthermore, the formation of a robust solid‐electrolyte interphase effectively stabilizes the Zn anode. As a result, the ternary water‐deficient electrolyte enables highly reversible Zn plating/stripping with low polarization under ambient conditions, while also delivering excellent low‐temperature performance. Enhanced kinetics in Zn||PANI cells and consistent performance across both coin cells and printed energy devices highlight the strong potential of this water‐deficient eutectic electrolyte for scalable zinc‐ion energy storage.

## Graphical abstract

A water‐deficient ternary eutectic electrolyte employs weakly coordinating solvents to decouple strong solvation interactions, constructing a loose and dynamic solvation network. This optimized solvation structure accelerates Zn^2+^ transport and interfacial kinetics, promotes robust SEI formation, and ultimately enables highly stable and reversible Zn plating/stripping.

## 1 Introduction

Zinc metal batteries have emerged as promising next‐generation energy storage systems due to their low cost, abundant availability, and high theoretical specific capacity. However, in conventional aqueous electrolytes, zinc metal anodes are thermodynamically unstable, which severely limits their electrochemical reversibility and practical applicability [1, 2]. During cycling, zinc anodes suffer from severe parasitic reactions, including the hydrogen evolution reaction (HER) and the formation of passivating byproducts [3, 4, 5]. These processes not only consume active zinc and electrolyte, resulting in low Coulombic efficiency, but also hinder reversible Zn^2+^ plating/stripping and exacerbate interfacial polarization [6, 7, 8]. Moreover, non‐uniform electric field distribution and restricted ion transport promote the formation of zinc dendrites, whose persistent growth compromises interfacial integrity and may trigger internal short circuits [9, 10]. The aforementioned issues collectively lead to rapid capacity decay, poor cycling stability, and safety concerns, representing critical bottlenecks for the large‐scale deployment of zinc metal batteries.

To mitigate thermodynamic instability and severe interfacial side reactions of zinc anodes in aqueous systems, attention has increasingly focused on water‐lean electrolytes, among which eutectic electrolytes have attracted considerable interest due to their unique physicochemical properties [11, 12, 13]. Typically constructed through strong interactions between zinc salts and hydrogen‐bond donors/acceptors, eutectic electrolytes exhibit low vapour pressure, wide electrochemical stability windows, and tunable solvation structures, enabling effective suppression of HER, reduced water activity, and enhanced interfacial stability of zinc metal [14, 15]. Consequently, eutectic electrolytes are regarded as promising alternatives for achieving highly stable zinc metal anodes. Herein, eutectic electrolytes are classified into three categories: (i) nonaqueous eutectic electrolytes, containing no free water; (ii) water‐rich eutectic electrolytes, with externally added water as the solvent; and (iii) water‐deficient eutectic electrolytes, where water is added only as crystallization water from the eutectic components without additional solvent incorporation.

In nonaqueous eutectic electrolytes, the strong intermolecular interactions inherent in the eutectic systems inevitably result in high viscosity and relatively low ionic conductivity, leading to sluggish ion transport kinetics and pronounced ohmic and interfacial polarization during battery operation [16]. This kinetic limitation remains a key challenge that must be addressed to advance eutectic electrolytes toward practical applications. In eutectic electrolyte systems, the controlled introduction of water has been widely adopted as an effective strategy to reduce viscosity, thereby improving Zn^2+^ mass transport and alleviating polarization [17, 18, 19]. As a result, hydrated eutectic electrolytes have been extensively explored for performance optimization. However, the influence of water content on eutectic electrolytes is difficult to define precisely: excessive water can weaken or even disrupt eutectic interactions, causing the system to gradually transition from a eutectic structure to a conventional mixed salt solution and, consequently, undermining the interfacial stabilization advantages of eutectic electrolytes. More critically, the incorporation of water inevitably increases the electrolyte's electrochemical activity, intensifying HER and other parasitic reactions, thereby accelerating the degradation of zinc anodes [17]. To overcome the intrinsic drawbacks of water solvent, certain low‐viscosity organic solvents have been employed to partially replace water and enhance ion transport; however, their inherent toxicity, stringent production requirements, and limited scalability substantially hinder further development. In addition, zinc salts containing Lewis‐basic halide or fluorinated anions, such as Zn(TFSI)_2_, Zn(OTF)_2_, Zn(ClO_4_)_2_, and ZnCl_2_, are frequently selected as zinc sources in eutectic electrolytes to construct stable solvation environments [20, 21, 22, 23]. Nevertheless, the high cost, strong corrosivity, and poor environmental compatibility of these salts pose significant challenges for large‐scale energy storage applications. Therefore, the development of novel eutectic electrolyte systems that simultaneously achieve moderate viscosity, high interfacial stability, and cost‐effectiveness is of paramount importance for enabling the scalable deployment of zinc‐ion energy storage devices.

**FIGURE 1:**
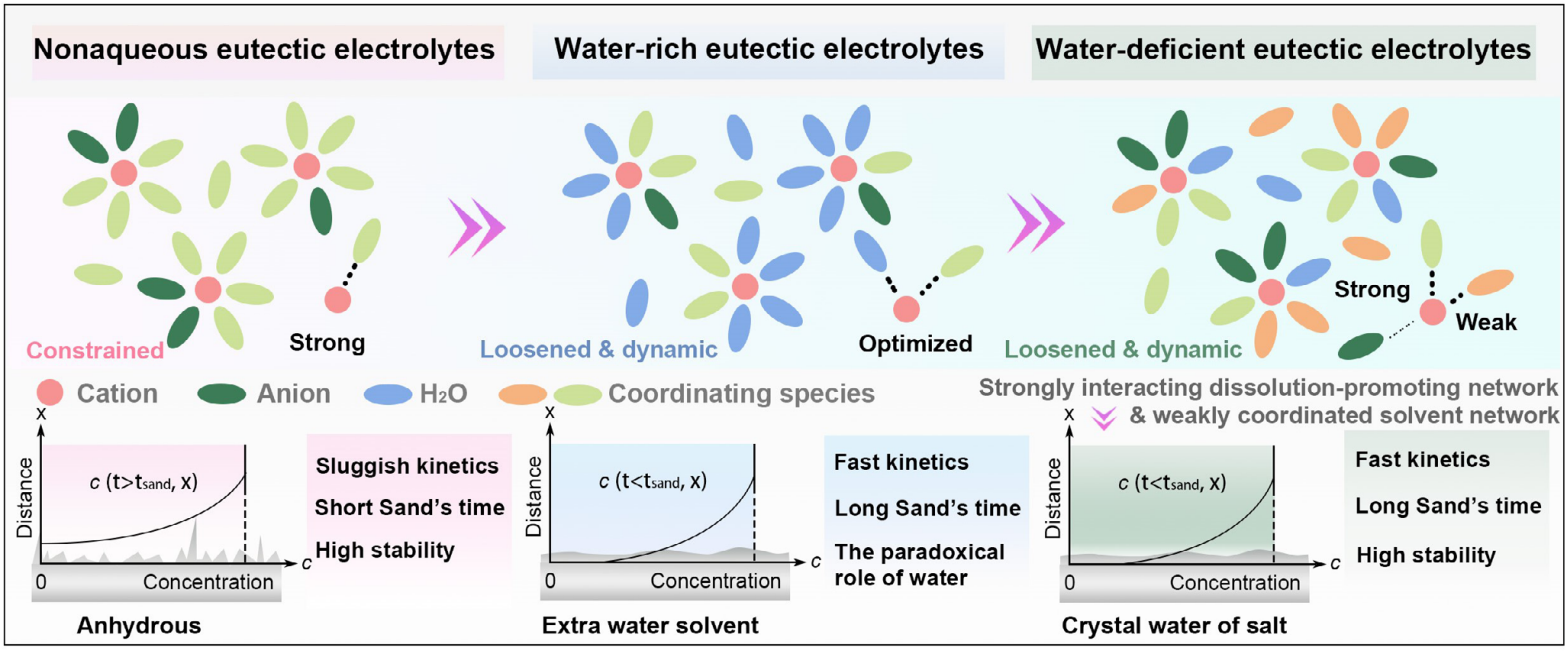
Mechanistic illustration of ternary water‐deficient eutectic solvents and comparison with nonaqueous and water‐rich eutectic systems.

Based on the above considerations, we design a low‐cost ternary eutectic electrolyte composed of an inexpensive zinc salt (ZnSO_4_·7H_2_O) as the zinc source and two industrially scalable solvents, ethylene glycol (EG) and methanol (MOH), without the intentional addition of free water. In this system, the crystal water associated with the zinc salt acts as a Lewis acid, while the two simple alcohols serve as Lewis bases. EG, as a strongly interacting solvent, promotes the formation of a robust solvation network and ensures efficient dissolution of ZnSO_4_·7H_2_O. Meanwhile, MOH, functioning as a weakly coordinating co‐solvent, modulates the strong Zn^2+^‐EG interactions and induces a more labile and dynamically exchanging solvation environment. This restructuring facilitates enhanced anion participation and the formation of size‐confined aggregates, thereby constructing a weakly coordinated solvent network (Figure 1). Compared with binary eutectic electrolytes, the ternary system exhibits enhanced ion‐transport kinetics and reduced energy barriers, leading to significantly improved electrochemical performance. More importantly, compared with conventional aqueous electrolytes, the optimized solvation structure promotes the formation of a stable solid‐electrolyte interphase (SEI), enabling highly reversible zinc plating/stripping behaviour. Notably, the optimized ternary eutectic electrolyte delivers polarization voltages comparable to those of aqueous electrolytes, despite the absence of excess water under ambient conditions. Furthermore, the electrolyte enables stable cycling for over 3500 h at −20°C, demonstrating outstanding anti‐freezing performance and robustness at low temperatures. Using this electrolyte, pouch cells fabricated with screen‐printed electrodes maintain favorable capacity retention of 98.46% over 1300 h even at low temperatures, highlighting its strong potential for scalable, low‐cost zinc‐ion energy storage applications.

## 2 Results and Discussion

EG and MOH, both bearing ─OH functional groups, are widely used solvents that can act as effective hydrogen‐bond donors and have demonstrated excellent solvation capabilities in various salt systems (such as Zn(OTF)_2_, Zn(BF_4_)_2,_ and ZnCl_2_) [23, 24, 25, 26, 27]. Importantly, both alcohols are produced on an industrial scale at low cost and high availability, rendering them particularly attractive for the construction of scalable, economically viable electrolytes. To minimise the detrimental influence of water on the thermodynamic stability of zinc metal and to preserve the integrity of the eutectic structure, no external water is introduced; Instead, only metal salts containing intrinsic crystallization water are considered. Accordingly, ZnSO_4_·7H_2_O, one of the most cost‐effective zinc salts, is selected as the zinc source (Cost comparison shown in Figure S1). Although anhydrous zinc salts may, in principle, reduce electrolyte‐induced corrosion of the zinc anode, the absence of sufficient hydrogen‐bond acceptors in such systems severely hampers salt dissolution and prevents the formation of a stable eutectic electrolyte, as evidenced in Figure S2 [28, 29]. An aqueous 2 mol L^−1^ ZnSO_4_ solution is selected as the base concentration, referred to as ZS, and the binary electrolyte ZnSO_4_·7H_2_O/EG is discussed as ZE. Considering that methanol not only participates in constructing the eutectic structure but also modulates the solvent network, thereby lowering the electrolyte viscosity, a series of electrolytes denoted as ZEMX. Here, X denotes the volume parts of MOH, corresponding to a volume ratio of MOH to EG of X: (10 – X), where X = 1, 3, 5, and 7.

**FIGURE 2:**
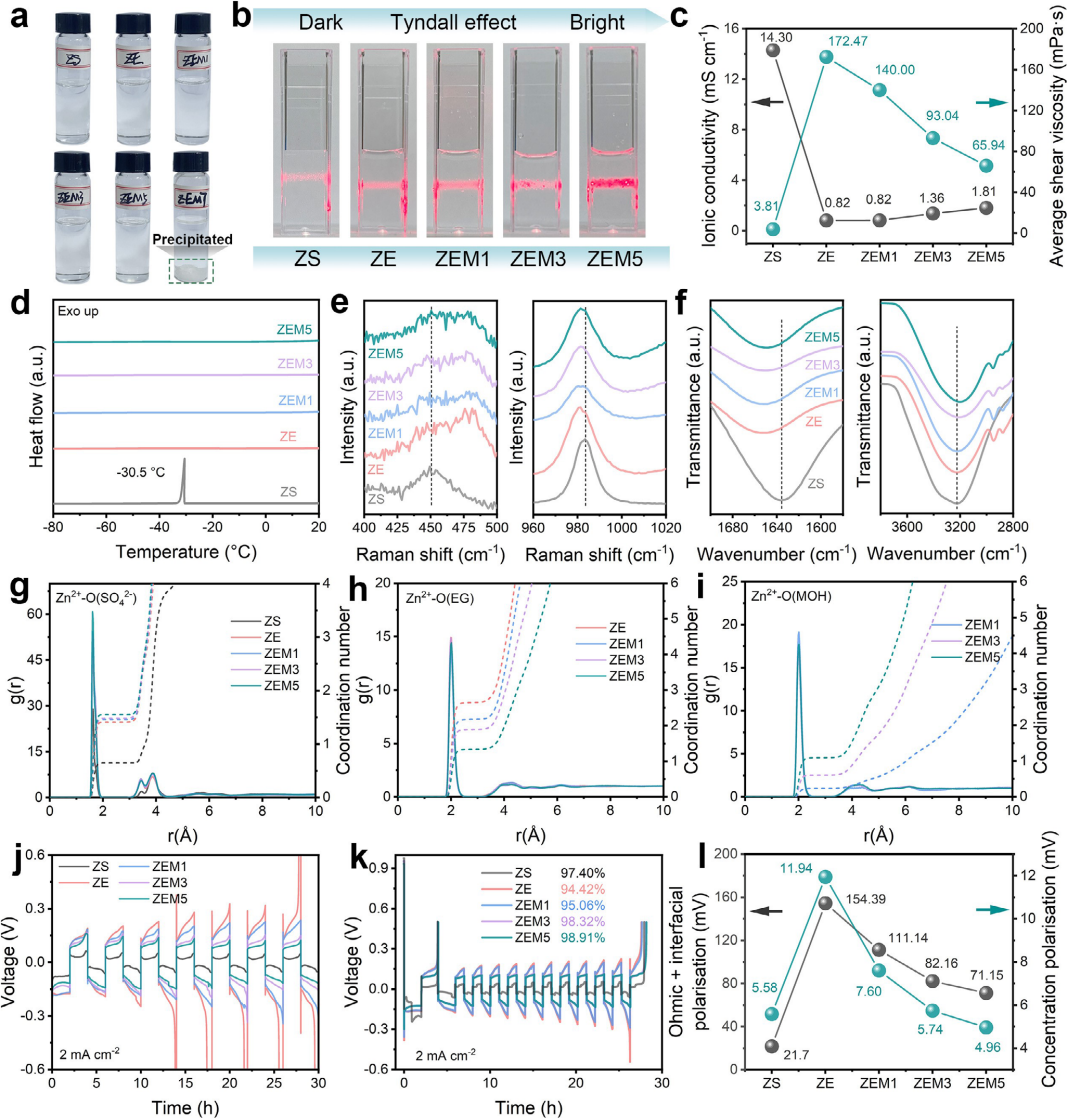
(a) Optical images of different electrolytes after one month of storage. (b) Tyndall effect of different electrolytes. (c) Ionic conductivity and viscosity of different electrolytes. (d) DSC, (e) Raman and (f) FTIR of different electrolytes. RDF for (g) Zn^2+^‐O(SO_4_^2−^), (h) Zn^2+^‐O(EG) and (i) Zn^2+^‐O(MOH). (j) Local voltage evolution during cycling. (k) CE curves and (l) Separation polarization curves of different electrolytes.

The basic physical properties of the electrolytes are first investigated. After storing the prepared binary eutectic electrolyte with EG as the solvent at room temperature for one month, it remains clear (Figure 2a). As the proportion of MOH in the solvent increases, the ZnSO_4_ in the ZEM5 electrolyte remains well dissolved. However, when the MOH proportion reaches 70% (ZEM7), clear precipitation of the inorganic salt is observed. Additionally, as shown in Figure 2b, the Tyndall effect intensifies from ZS to ZE and then to ZEM5, indicating significant changes in the ion clusters within the electrolyte [30]. The conductivity and viscosity of ZS and the eutectic electrolytes are presented in Figure 2c and Figures S3 and S4. ZS electrolyte exhibits the highest conductivity at 14.30 mS cm^−1^, while the conductivities of ZE and ZEM1 are similar, at just 0.82 mS cm^−1^. ZEM3 has a conductivity of 1.36 mS cm^−1^, and ZEM5 shows the highest conductivity among the eutectic systems at 1.81 mS cm^−1^. The aqueous electrolyte ZS has the lowest viscosity at 3.81 mPa·s, while ZE exhibits the highest viscosity at 172.47 mPa·s. The viscosities of ZEM1, ZEM3, and ZEM5 are 140.00, 93.04, and 65.94 mPa·s, respectively. These results show that, within the eutectic electrolyte system, as the MOH content increases, conductivity gradually improves, and viscosity decreases. Differential scanning calorimetry (DSC) analysis clearly shows the thermal behaviours of different electrolytes (Figure 2d). ZS electrolyte has a freezing point of −30.5°C, while ZE and ZEMX show no distinct thermal peaks. This behaviour arises from the strong hydrogen bonding between organic solvent molecules and water molecules, which competes with and disrupts water–water hydrogen‐bond interactions, thereby suppressing the formation of an ordered hydrogen‐bond network and maintaining the liquid phase, indicating the superior anti‐freezing capability of the eutectic electrolyte (Figure S5) [31]. Additionally, contact angle measurements reveal that the eutectic electrolytes exhibit significantly smaller contact angles than ZS, as shown in Figure S6. As the MOH component increases, the contact angle decreases, indicating better wetting properties and enhanced zinc affinity.

Raman spectroscopy reveals substantial changes in sulfate coordination as the solvent system is varied (Figure 2e). Compared with aqueous ZS, the SO_4_^2^^−^ bending vibration centred at ∼450 cm^−1^ exhibits pronounced broadening and splitting in the 425–500 cm^−1^ region in eutectic electrolytes, suggesting more diverse Zn^2+^‐O(SO_4_^2^^−^) coordination environments and the formation of multiple ion‐pair species [32]. In addition, the characteristic SO_4_^2^^−^ stretching band at 983.2 cm^−1^ undergoes a clear redshift, indicating reduced vibrational freedom of sulfate anions and strengthened electrostatic interactions between Zn^2+^ and SO_4_^2^^−^, thereby enhancing the coordination ability of sulfate [33]. Infrared spectra further show that, with the evolution of the solvent system, the H‐O‐H bending vibration at 1636.3 cm^−1^ exhibits a noticeable blueshift, while the broad O─H stretching band centred at 3222.5 cm^−1^ shifts to lower wavenumbers (Figure 2f). These spectral changes indicate significant alterations in the local coordination and hydrogen‐bonding environments of the crystallization water and solvent molecules, suggesting a reconstructed solvation environment in the eutectic electrolytes [34, 35].

Molecular dynamics (MD) simulations are performed to obtain radial distribution functions (RDF) to further elucidate the solvation structure of Zn^2+^ ions. Coordination information in different electrolyte systems is quantitatively extracted from the RDF analysis. As shown in Figure 2g, the coordination number of Zn^2+^‐SO_4_^2^^−^ gradually increases from 0.65 in ZS to 1.41 in ZE and further to 1.55 in ZEM5. In contrast, the coordination number of Zn^2+^‐O(EG) decreases from 2.65 in ZE to 1.34 in ZEM5, whereas the coordination number of Zn^2+^‐O(MOH) monotonically increases from 0.24 in ZEM1 to 1.09 in ZEM5 (Figure 2h,i). With increasing MOH content, the decrease in Zn^2+^‐EG coordination and the concomitant increase in Zn^2+^‐MOH coordination clearly indicate a pronounced competitive coordination effect between EG and MOH molecules. Notably, compared with the aqueous electrolyte, the average coordination number of water molecules surrounding Zn^2+^ drastically decreases from 5.35 in ZS to approximately 2 in the eutectic electrolytes (Figure S7). Furthermore, proton nuclear magnetic resonance spectroscopy verifies the effect of MOH incorporation on the binary eutectic electrolyte and provides further evidence that MOH directly participates in the Zn^2+^ solvation structure (Figure S8).

The electrochemical behaviour of different electrolytes is preliminarily evaluated using Zn||Zn symmetric cells. ZS exhibits the smallest polarization voltage at 2 mA cm^−2^, whereas ZE shows the largest polarization in the voltage–time profiles Figure 2j. With increasing MOH content, the polarization voltages of ZEM1, ZEM3, and ZEM5 decrease monotonically, and ZEM5 delivers the longest cycling lifetime among the eutectic electrolytes (Figure S9). Coulombic efficiency, evaluated using the Aurbach method, is 97.4% for ZS, while ZEM5 achieves the highest value of 98.91% (Figure 2k), demonstrating its superior reversibility. Furthermore, the galvanostatic intermittent titration technique is employed to obtain different polarization contributions (Figure 2l and Figure S10). The eutectic electrolytes exhibit greater ohmic and interfacial polarization than the aqueous electrolyte, attributed to their lower ionic conductivity under higher viscosity. Importantly, both ohmic and interfacial polarization gradually decrease with increasing MOH content. A similar trend is observed for concentration polarization; notably, ZEM5 unexpectedly exhibits a smaller concentration polarization than ZS. Based on this preliminary evaluation, ZS, ZE, and ZEM5 are selected for more in‐depth investigation.

**FIGURE 3:**
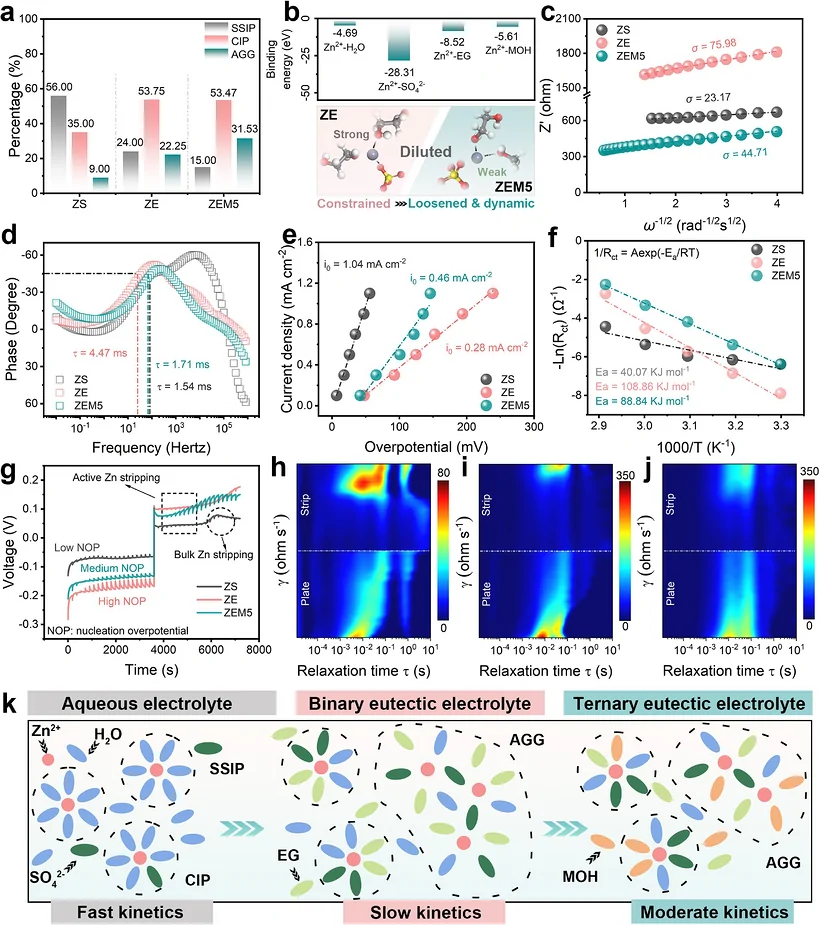
(a) Percentage of solvation types in different electrolyte systems. (b) Binding energy. (c) The Warburg coefficient of different electrolytes in the low‐frequency region. (d) Bode plot. (e)Exchange current densities of different electrolytes. (f) Activation energies of different electrolytes. (g) Voltage‐time plots. DRT of (h) ZS, (i) ZE and (j) ZEM5 using operando EIS. (k) Schematic diagram illustrating the bulk electrolyte structures in three different electrolyte systems.

The possible ion pairs in ZS, ZE, and ZEM5 electrolyte systems are statistically analysed using MD simulations. According to the RDF analysis, competitive coordination leads to a change in the solvation structure, causing the electrolyte to gradually transition from a solvent‐dominated “ion‐solvent” structure to a salt‐dominated “ion‐ion” solvation structure [36]. This shift results in ion aggregation, forming ion clusters that may lead to higher viscosity and restricted ion diffusion. In aqueous electrolytes, a small amount of sulfate ions participates in the initial solvation structure. As shown in Figure 3a, the eutectic electrolyte induces a restructuring of the solvation environment, shifting the electrolyte from a predominance of solvent‐separated ion pairs (SSIP) to a structure dominated by contact ion pairs (CIP) and ion‐pair aggregate (AGG). The AGG structure is optimized in this process. The scattering curve of the eutectic electrolyte shows a lower q‐value, indicating an increase in the size of ion‐molecule coordination clusters (Figure S11). First‐principles calculations reveal that Zn^2+^ exhibits the strongest binding with SO_4_^2^^−^, dominated by electrostatic interactions. Among the examined solvent molecules, Zn^2+^ shows the highest binding energy toward EG and the weakest toward H_2_O, highlighting the critical role of solvent coordination strength in governing the solvation structure. The introduction of MOH, a weakly coordinating solvent, induces a ligand‐exchange‐driven solvation restructuring in the ternary system, effectively weakening the strong Zn^2+^‐EG interactions present in the binary eutectic. This transition shifts the electrolyte from a strongly coordinated, solvent‐dominated regime to a more labile and dynamically exchanging coordination environment. Consequently, a weakly coordinated solvent network is established, which promotes anion incorporation into the primary solvation sheath while suppressing extended ion bridging and long‐range aggregation. Combining the points previously mentioned, the Tyndall effect is most pronounced in ZEM5. It can therefore be concluded that in the eutectic system, ZEM5 contains numerous smaller solvation clusters, whereas ZE exhibits relatively larger ones [30]. In addition to the bulk electrolyte studies, differential capacitance measurements are conducted to investigate the electrode/electrolyte interface. ZS electrolyte shows the highest interface capacitance at 37.59 µF cm^−2^, ZE the lowest at 28.30 µF cm^−2^, and ZEM5 at 30.01 µF cm^−2^ (Figure S12). These results highlight a significant change in the double‐layer structure between aqueous and eutectic electrolytes. The eutectic system displays a thicker double‐layer structure, and the differences between ZE and ZEM5 may be attributed to size effects.

To decouple the intrinsic ion‐transport behaviour from Faradaic activation associated with battery‐type electrodes, hybrid capacitors are assembled to evaluate the kinetic response of different electrolytes. Electrochemical impedance spectroscopy (EIS), shown in Figure S13a, reveals that the interfacial resistance follows the trend ZS < ZEM5< ZE, consistent with the conductivity measurements (Figure S3). In contrast, the charge‐transfer resistance (R_ct_) highlights distinct interfacial reaction kinetics, following ZEM5< ZS < ZE, which suggests that the ternary eutectic electrolyte (ZEM5) establishes a more favorable interfacial environment for charge transport during the initial interface formation and facilitates faster interfacial ion exchange compared with the aqueous ZS and highly viscous ZE systems. This reduces the interfacial reaction barrier, thereby facilitating a smoother charge‐transfer process. In the low‐frequency region, the linear dependence of the real impedance (Z′) on *ω* ^−1/2^ (*ω*: angular frequency) allows extraction of the Warburg coefficient, which reflects the long‐range diffusion capability of Zn^2+^ (Figure 3c) [37]. The three different electrolytes exhibit distinct diffusion behaviour: ZS shows the smallest Warburg coefficient (23.17), ZEM shows an intermediate value (44.71), and ZE presents the largest (75.98). These results indicate that although Zn^2+^ diffusion is intrinsically slower in eutectic systems compared with aqueous ZS, the ZEM electrolyte system substantially alleviates the diffusion limitations observed in ZE. This improvement is attributed to the tailored solvation structure and moderated viscosity of ZEM, which together promote more efficient ion migration. The same result is obtained using the diffusion coefficient and MSD (Figures S13b and S14). Furthermore, the characteristic relaxation time (τ), operationally extracted from the Bode phase plot at a phase angle of ‐45°, reflects the characteristic timescale of the dominant ionic/interfacial response. Bode analysis yields τ values of 1.54 ms (ZS), 1.71 ms (ZEM), and 4.47 ms (ZE), indicating that ZEM substantially mitigates the sluggish relaxation behaviour of ZE and approaches the response level of the aqueous electrolyte (Figure 3d). Taken together, Rct reflects interfacial charge‐transfer kinetics, the diffusion coefficient characterises long‐range ion diffusion, and τ represents the dominant dynamic response associated with electrolyte‐governed ion transport in the cell. Their combined trends clearly show that the ternary eutectic ZEM electrolyte offers a well‐balanced transport environment with markedly improved kinetics over the ZE system and only slightly lower performance than aqueous ZS. This synergistic enhancement underscores the effectiveness of molecular‐level eutectic modulation in optimising ion transport and interfacial behaviour. Moreover, as depicted in Figure 3e, the ZS electrolyte demonstrates the highest exchange current density at 1.04 mA cm^−2^, whereas the ZE electrolyte shows the lowest value at 0.28 mA cm^−2^. ZEM5 eutectic electrolyte presents an intermediate value of 0.46 mA cm^−2^. This suggests that the zinc ion transfer and reaction kinetics are most efficient in the ZS electrolyte, owing to its superior conductivity and low viscosity. In contrast, the eutectic electrolyte ZE, characterised by lower conductivity, higher viscosity, and larger aggregate formations, exhibits the slowest kinetics. The optimization of aggregate structures in ZEM5 effectively narrows the gap between traditional eutectic and aqueous solvents, thereby substantially enhancing both ionic migration and reaction kinetics.

The activation energy for interfacial charge transfer, derived from the Arrhenius equation, reflects the energy barrier Zn^2+^ must overcome during desolvation and electron exchange at the electrode interface [38]. The aqueous electrolyte ZS exhibits a relatively low activation energy (Ea) of 40.07 kJ mol^−1^, whereas the eutectic electrolyte ZE shows a much higher Ea of 108.86 kJ mol^−1^, followed by an intermediate value of 88.84 kJ mol^−1^ for the ternary eutectic electrolyte ZEM5 (Figure 3f and Figure S15). These results indicate that ZS exhibits the fastest interfacial reaction kinetics, with the easiest desolvation process. On the other hand, the ZE electrolyte exhibits slower kinetics due to stronger solvation, requiring greater effort to desolvate. The ternary eutectic electrolyte shows moderate behaviour, suggesting that competitive coordination reduces kinetic resistance.

During deposition, significant nucleation differences are observed among the electrolytes. As illustrated in Figure 3g, both ZE and ZEM5 electrolytes have higher nucleation overpotentials, promoting the formation of dense zinc nuclei. In the time‐voltage curves, a distinct inflexion point is seen during the stripping stage for the ZS electrolyte, indicating bulk zinc dissolution and poorer reversibility [39, 40]. Further distribution‐of‐relaxation‐times (DRT) analysis provides deeper insights into the evolution of interfacial kinetic processes. Owing to its high ionic conductivity and low viscosity, the aqueous ZS electrolyte exhibits the fastest overall ionic/interfacial response, as reflected by minimal relaxation times across the examined frequency range (Figure 3h). However, the rapid kinetics are accompanied by uneven Zn deposition and stripping, ultimately resulting in inferior cycling stability. By contrast, ZE and ZEM5 display comparable relaxation time distributions across different kinetic regimes (Figure 3i,j), indicating similar underlying interfacial and diffusion‐related processes. Notably, the impedance response of ZEM5 gradually decreases in the mid‐ and low‐frequency regions during cycling, suggesting stable interfacial evolution and enhanced reversibility. In comparison, ZE initially exhibits a relatively high charge‐transfer‐related impedance that decreases rapidly, followed by pronounced features in the diffusion‐dominated region, indicative of increased interfacial heterogeneity and stronger diffusion limitations. Such behaviour is likely associated with the evolution of the interfacial structure during the initial deposition process. To minimise the influence of the initial Zn deposition layer, DRT analysis is further performed after 10 cycles (Figure S15). In the 1–10 s relaxation time range, the DRT peaks of ZE and ZEM5 nearly overlap, suggesting similar diffusion mechanisms. Nevertheless, the peak intensity and integrated area for ZE are significantly larger than those of ZEM5, indicating higher diffusion‐related impedance and more severe concentration polarization. During the stripping process, ZEM5 exhibits a smoother decay across all relaxation regimes, implying more uniform Zn stripping and a more reversible interface with reduced diffusion constraints. Overall, although ZE and ZEM5 share similar kinetic processes, ZE suffers from more pronounced diffusion limitations, whereas ZEM5 demonstrates improved diffusion kinetics and suppressed concentration polarization, highlighting the kinetic advantages of the ternary eutectic electrolyte over the binary eutectic system. Based on the above discussion, as shown in Figure 3k, differences in the solvation environment lead to kinetic variations in the behaviour of zinc ions in bulk electrolytes, allowing ternary eutectic electrolytes to emerge as contenders capable of competing with aqueous electrolytes. Meanwhile, CE measurements conducted at a current density of 1 mA cm^−2^ show that ZEM5 delivers an average Coulombic efficiency of 99.71% (Figure S17), which is higher than that of ZE (97.22%) and ZS (99.05%). Therefore, ZEM5 is selected as the modified electrolyte for further research.

**FIGURE 4:**
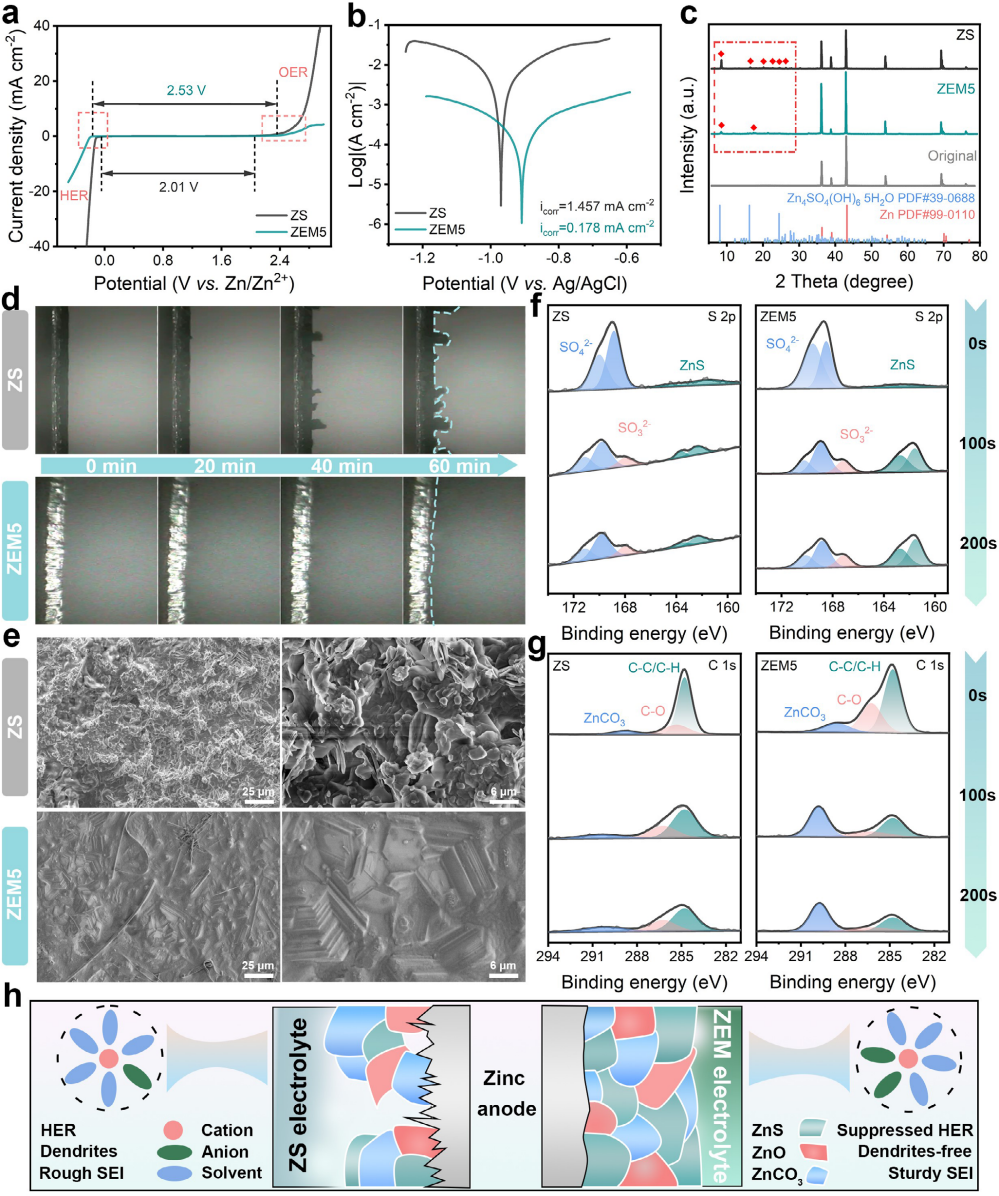
(a) LSV curves of ZS and ZEM5 electrolytes. (b) Tafel plots of ZS and ZEM5 electrolytes. (c) XRD patterns of Zinc foils after cycling. (d) In situ optical microscopy images of different electrolytes. (e) SEM images after cycling with different electrolytes at 1 mA cm^−2^. (f) S 2p and (g) C 1s XPS spectra of the zinc anode with Ar^+^ sputtering. (h) Illustration of the effect of different electrolytes.

To assess the practical impact of different electrolytes on the zinc metal anode, a detailed comparison of various electrolyte systems is presented. Linear sweep voltammetry shown in Figure 4a reveals that the aqueous electrolyte ZS exhibits a relatively narrow electrochemical stability window of 2.01 V. In stark contrast, the eutectic electrolyte displays a significantly expanded stability window of 2.53 V, accompanied by a pronounced negative shift in the onset potential for hydrogen evolution and a positive shift for oxygen evolution. These shifts indicate that the reduction in coordinated water and the disruption of the hydrogen‐bonded network between water molecules enhance the electrochemical stability of the eutectic electrolyte. Moreover, Tafel curve analysis provides crucial insights into the corrosion kinetics of zinc foil in these different electrolyte systems. As illustrated in Figure 4b, the eutectic electrolyte exhibits a remarkably low corrosion current of only 0.178 mA cm^−2^, while the corrosion current for the ZS electrolyte is substantially higher, at 1.427 mA cm^−2^. Notably, the ZEM5 electrolyte system displays a more positive corrosion potential relative to ZS, indicating a lower susceptibility to corrosion [41]. This is further supported by static corrosion tests, in which zinc foil is immersed in ZS and ZEM5 electrolytes for 1 week. SEM images reveal that the zinc foil develops a distinctly irregular surface morphology after immersion in the ZS electrolyte, indicative of significant by‐product formation (Figure S18). In contrast, the surface of the zinc foil in the ZEM5 electrolyte remains smooth and flat, resembling the original morphology, free from any by‐product accumulation. X‐Ray Diffraction (XRD) patterns further corroborate the superior stability of the eutectic electrolyte (Figure S19). When analysing the surface characteristics of zinc foil cycled under identical conditions in different electrolytes, the XRD data clearly show that the ZS electrolyte is associated with more pronounced peaks for by‐products, such as basic zinc sulfate, while the ZEM5 electrolyte presents only minimal by‐product peaks, signifying a substantial suppression of parasitic reactions during the deposition/stripping process (Figure 4c).

To understand the nucleation behaviour of zinc ions, asymmetric batteries are assembled for detailed investigation. The eutectic electrolyte, ZEM5, exhibits a higher nucleation overpotential than the ZS electrolyte, as shown in Figure S20, promoting the growth of compact, fine nuclei and thereby fostering a more uniform, dense deposition process. Chronoamperometry measurements further reveal significant differences in the diffusion characteristics of zinc ions in these systems (Figure S21). The eutectic system exhibits a lower, more stable current response over a brief period, as solvent molecules form an adsorption layer that increases the lateral diffusion barrier, thereby facilitating vertical ion transport under the electric field. This suggests a transition toward three‐dimensional diffusion [19]. Conversely, the water‐based electrolyte exhibits increasing current over time, indicative of uncontrolled dendrite growth and a two‐dimensional diffusion process associated with an expanding effective surface area. The deposition process of Zn^2+^ is further investigated using in situ optical microscopy. As shown in Figure 4d, the surface of the zinc foil in the ZS electrolyte progressively develops distinct dendritic growth patterns with increasing deposition time. In stark contrast, the surface of the zinc foil in the ZEM5 electrolyte remains smooth and flat throughout the deposition process, clearly highlighting the superior deposition morphology in the eutectic electrolyte. SEM analysis of zinc foil after 30 cycles (Figure 4e) further confirms this observation, with the ZS electrolyte resulting in a rough, dendrite‐laden surface, while the ZEM5 electrolyte yields a smooth, uniformly deposited structure. XRD analysis of the zinc foil after various cycle counts reveals a consistent increase in the relative texture coefficient of the (002) crystal plane, providing strong evidence that zinc ions predominantly deposit along the (002) plane (Figure S22). This orientation significantly reduces the risk of dendrite formation and potential short circuits, thereby improving the overall cycle stability.

X‐ray photoelectron spectroscopy (XPS) is used to analyse the chemical composition of zinc metal foil after electrochemical cycling. In the S 2p spectra, weak peaks corresponding to ZnS are detected on zinc foil cycled in both water‐based and eutectic electrolytes (Figure 4f). The intensity of these ZnS peaks increases with sputtering time, with a more pronounced increase in the ZEM5 electrolyte than in the ZS electrolyte. The formation of ZnS is attributed to the reduction of SO_4_^2^^−^ anions at the electrode surface, facilitated by electron transfer during the electrochemical process [42]. This behavior is linked to the marked increase in CIP content from ZS to ZEM5, which leads to a greater number of anions being induced to the electrode surface. These anions gain electrons, thereby forming a stable SEI. Similarly, the C 1s spectra reveal peaks corresponding to ZnCO_3_ on the Zinc foil surface (Figure 4g). With increasing sputtering depth, weak ZnCO_3_‐related peaks can still be observed on the Zinc foil using the ZS electrolyte. These signals are likely attributed to the facile reaction of surface ZnO/Zn(OH)_2_ species with atmospheric CO_2_, leading to the formation of ZnCO_3_ species [43, 44]. Meanwhile, the Zn─O component in the O 1s spectrum (Figure S23a) can be attributed to Zn‐containing species, including ZnCO_3_ and ZnO [45]. Zn Auger electron spectroscopy, illustrated in Figure S23b, supports these findings, showing that, with increasing sputtering time, the zinc foil cycled in the ZEM5 electrolyte maintains a stable composition, always associated with zinc compounds. Moreover, time‐of‐flight secondary ion mass spectrometry analysis further corroborates the XPS results, confirming the formation of a compact and homogeneous SEI layer on the Zinc foil in the ZEM5 electrolyte (Figure S23c). This robust interfacial layer effectively suppresses parasitic reactions and remains stable during electrochemical cycling. These results demonstrate that the ZEM5 eutectic electrolyte promotes the formation of a durable protective SEI, thereby enhancing the interfacial stability of the Zn anode. The underlying mechanism can be summarised as shown in Figure 4h: when the solution composition changes, the reduced water content in the solvation structure effectively suppresses hydrogen evolution reactions, thereby enhancing electrochemical stability. In the eutectic system, increasing CIP concentration increases the participation of anions in the solvation structure, facilitating their reduction at the interface. This process forms a solid SEI that effectively protects the zinc anode from corrosion and degradation. Additionally, the solvent in the eutectic system aids in guiding the uniform deposition of zinc ions by promoting interface adsorption on the anode, further reducing the risk of dendrite formation and short‐circuiting.

**FIGURE 5:**
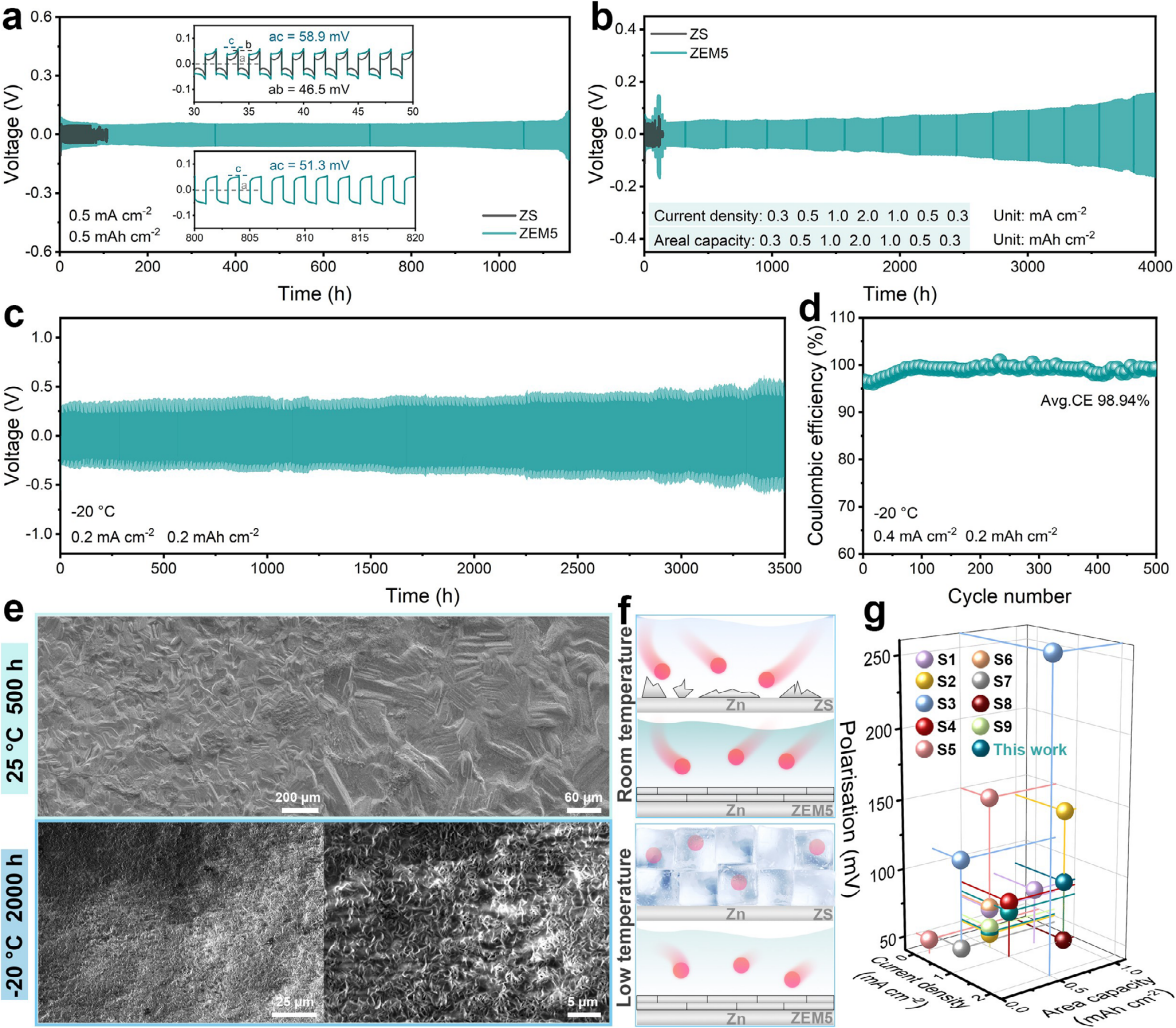
(a) Cycle performance of different electrolytes at 0.5 mA cm^−2^. (d) Rate performance. (c) The cycle performance of ZEM5 at −20°C. (d) CE of ZEM5 at −20°C. (e) SEM images of Zinc foil after cycling. (f) Diagram showing the influence of electrolytes at different temperatures. (g) Comparison between ZEM5 and previously reported eutectic electrolytes (Table S1).

Subsequently, the practical applicability of the electrolyte is evaluated using Zn||Zn symmetric cells. At a current density of 0.5 mA cm^−2^, the symmetric cell employing the ZEM5 electrolyte delivers a stable cycling life of nearly 1160 h, shown in Figure 5a, significantly outperforming the ZS electrolyte–based cell (72 h). The polarization voltage of Zn||Zn symmetric cell using the eutectic electrolyte at 0.5 mA cm^−2^ is comparable to that of the aqueous ZS electrolyte, indicating favorable interfacial reaction kinetics. As the current density increases to 1 mA cm^−2^, the Zn||Zn cell with the ZS electrolyte exhibits voltage fluctuations within 100 h and suffers an obvious short circuit at approximately 130 h. In sharp contrast, the ZEM5‐based symmetric cell delivers durable cycling performance for 885 h (Figure S24a). Moreover, the ZEM5‐based symmetric cell exhibits a prolonged cycling life of more than 3000 h (1.0 mA cm^−2^ and 0.5 mAh cm^−2^), whereas the ZS‐based cell fails within 250 h (Figure S24b). In addition, ZEM5 exhibits superior rate capability; after switching to a lower current density of 0.3 mA cm^−2^, the symmetric cell continues to operate stably for over 3800 h, while the ZS electrolyte leads to premature short‐circuit failure (Figure 5b). Beyond room‐temperature performance, the eutectic electrolyte endows the symmetric cells with excellent anti‐freezing capability. At −20°C, the symmetric cell delivers a cycling lifetime of 3500 h at 0.2 mA cm^−2^ and also maintains stable operation at 0.5 mA cm^−2^ (Figure 5c and Figure S25). Furthermore, an average Coulombic efficiency of 98.94% is retained after 500 cycles at −20°C, highlighting the excellent reversibility of Zn plating/stripping under low‐temperature conditions (Figure 5d). In addition, the surface morphologies of Zinc foils harvested after cycling at different temperatures are systematically investigated. Specifically, the Zinc foil collected after 500 h of cycling at a current density of 1 mA cm^−2^ exhibits a uniform and compact deposition morphology, indicating the effective regulation of Zn plating/stripping behaviour by the ZEM5 electrolyte (Figure 5e). Notably, even after prolonged cycling for 2000 h at −20°C, the zinc foil surface remains smooth and homogeneous. Based on the above results, as illustrated in Figure 5f, ZEM5 delivers superior cycling stability and highly uniform Zn deposition at room temperature, markedly outperforming its aqueous counterpart. Notably, it retains stable operation under low‐temperature conditions, whereas the symmetric cell based on the aqueous electrolyte becomes inoperative. Furthermore, the ternary eutectic electrolyte exhibits compelling competitiveness relative to previously reported eutectic systems (Figure 5g).

**FIGURE 6:**
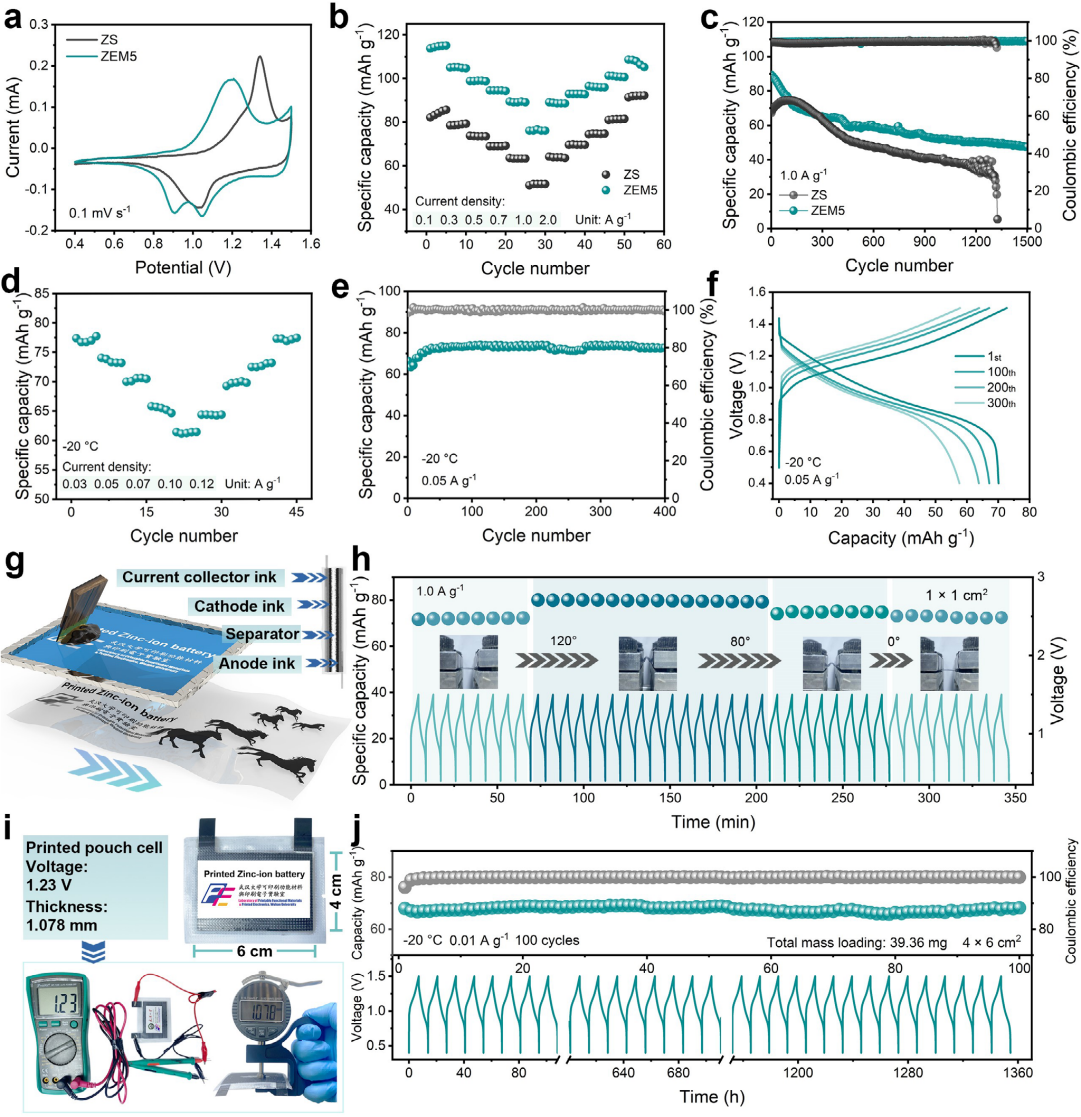
(a) CV curves of Zn||PANI with ZS and ZEM5 electrolytes. (b) Rate performance. (c) Cycle performance at 1.0 A g^−1^. (d) Rate performance at −20°C. (e) Cycle performance at −20°C and a current density of 0.05 A g^−1^. (f) Voltage profiles of different cycles at 0.05 A g^−1^. (g) Schematic illustration of the fabrication process of screen‐printed electrodes and device. (h) The performance of printed Zn||PANI at different bending conditions. (i) Optical images of the open‐circuit voltage and dimensions of printed Zn||PANI pouch cell. (j) Cycle performance of printed Zn||PANI pouch cell at −20°C and 0.01 A g^−1^.

To evaluate the effectiveness of the electrolyte, PANI is synthesised (Figure S26), and Zn||PANI batteries are assembled and systematically investigated. EIS in Figure S27 reveals that the cell employing ZEM5 exhibits a markedly lower charge‐transfer resistance, indicating accelerated interfacial kinetics [46]. Consistently, CV curves show a larger enclosed area for ZEM5, suggesting enhanced electrochemical reactivity and higher charge‐storage capability (Figure 6a). Compared with the ZS electrolyte, the reduction peak at 1.04 V splits into two distinct peaks at 0.90 and 1.00 V in ZEM5 electrolyte, indicating that the stepwise redox processes of PANI are better resolved as the reaction kinetics improve [47]. Meanwhile, the oxidation peak at 1.34 V shifts to a lower potential, suggesting that the electrode reaction polarization is effectively reduced, thereby enhancing the redox reaction kinetics. Therefore, Zn||PANI batteries with the ZEM5 electrolyte deliver significantly higher capacities (Figure 6b). Specifically, at current densities ranging from 0.1 to 2.0 A g^−1^, the ZEM‐based battery exhibits capacities of 108.67, 101.25, 96.53, 92.94, 89.17, and 71.11 mAh g^−1^, respectively, which are markedly higher than those obtained with the ZS electrolyte (91.42, 81.00, 74.44, 69.61, 64.17, and 51.67 mAh g^−1^). At a current density of 1 A g^−1^, the ZEM5‐based batteries maintain a capacity of 47.70 mAh g^−1^ after 1500 cycles, whereas ZS‐based batteries exhibit severe degradation and failure after 1200 cycles (Figure 6c). Moreover, the ZEM5‐based batteries retain 61.74% of its initial capacity at 0.5 A g^−1^ after 500 cycles, substantially outperforming the ZS counterpart (26.76%) shown in Figure S28. These results clearly demonstrate the improved cycling stability enabled by the eutectic electrolyte. Benefiting from the excellent anti‐freezing capability of ZEM5, the electrochemical performance of Zn||PANI full batteries is further evaluated at −20°C. As shown in the corresponding electrochemical profiles, the ZEM5‐based batteries maintain favorable rate capability even under subzero conditions (Figure 6d). Specifically, capacity retention reaches 90.31% after 1000 cycles at 0.10 A g^−1^ and remains as high as 97.83% after 400 cycles at 0.05 A g^−1^ (over 45 days), highlighting the outstanding low‐temperature electrochemical stability of the ZEM5 electrolyte (Figure S29 and Figure 6e,f).

Screen printing, as a scalable and low‐cost fabrication technique compatible with flexible substrates, has attracted increasing attention and experienced rapid development in the field of energy storage and conversion in recent years [48]. This technique enables the controlled deposition of electrode materials onto substrates by formulating them into printable inks, and electrodes with various patterns can be readily obtained through rational pattern design (Figure 6g). First, zinc powder and PANI are formulated into functional printable inks with favorable rheological properties, whose viscosity and shear‐thinning behaviour satisfy the requirements of the screen‐printing process (Figure S30). The excellent printability of the inks enables the fabrication of finely patterned electrodes to meet practical application requirements (Figure S31). Meanwhile, the thicknesses of the printed cathode and anode layers are approximately 83 and 56 µm, respectively (Figure S32). SEM results show that the screen‐printed electrodes exhibit dense surfaces with a uniform distribution of active material (Figure S33). Electrochemical measurements shown in Figure S34 further demonstrate that the CV curves of the printed batteries show highly consistent redox features and electrochemical responses compared with those of the corresponding coin cells, indicating that the screen‐printing process does not significantly impair the intrinsic electrochemical activity of the active materials. These results collectively verify the feasibility and application potential of screen printing for constructing high‐performance, printable, and flexible energy storage devices.

Systematic electrochemical performance evaluations of the printed Zn||PANI batteries further confirm the feasibility and stability of the fully printed devices. The printed Zn||PANI batteries still maintain good rate performance over a current density range of 0.1–2.0 A g^−1^ (Figure S35). At room temperature, the printed batteries operate stably for 350 cycles at of 1.0 A g^−1^, delivering a capacity retention of 74.20%, which demonstrates favorable cycling stability and suggests that the screen‐printing process does not compromise the electrochemical activity of the electrodes or the interfacial stability (Figure S36). In addition, the electrochemical performance of the printed batteries at −20°C is further investigated. The printed batteries still exhibit excellent rate capability and cycling performance (Figures S37–S39). Specifically, a capacity retention of 74.31% is maintained after 350 cycles at 0.05 A g^−1^, while 63.01% of the initial capacity is preserved after 850 cycles at a higher current density of 0.10 A g^−1^. These results indicate that the printed batteries maintain stable electrochemical reaction kinetics at low temperatures, highlighting their potential for applications in extreme environments.

Furthermore, the electrochemical performance of the printed batteries under different bending angles is evaluated (Figure 6h). At a current density of 1.0 A g^−1^, the battery capacity remains generally stable. After the initial cycles, a slight increase in capacity is observed as the bending angle increases to 120°, and the capacity remains stable during subsequent cycling. This phenomenon is attributed to enhanced interfacial contact between the electrodes and the electrolyte induced by bending. When the bending angle decreases to 80°, the capacity slightly decreases but remains stable. Upon recovery to the initial flat state, the capacity nearly returns to its original level, demonstrating excellent structural integrity and electrochemical stability of the printed batteries under repeated bending conditions. These results further confirm the feasibility of printed batteries as flexible, printable energy storage devices. Beyond micro‐scale printed batteries, printed Zn||PANI pouch cells are also successfully fabricated via screen printing (Figure 6i). Considering the excellent low‐temperature performance of the electrolyte, the −20°C electrochemical performance of the printed pouch cells is further investigated. At a low current density of 0.01 A g^−1^, the printed pouch cells cycle stably for 100 cycles (>1300 h) while maintaining high‐capacity stability of 98.46%, indicating that screen‐printed pouch cells can also deliver high performance under low‐temperature conditions (Figure 6j). As a proof of concept, either three micro‐scale printed batteries connected in series and frozen at low temperatures or two printed pouch cells connected to a timer can stably power devices, further demonstrating the practical application potential of printed zinc‐ion batteries in portable electronic devices (Figure S40).

## 3 Conclusion

In summary, an advanced low‐cost ternary eutectic electrolyte composed of ZnSO_4_·7H_2_O, ethylene glycol, and methanol is designed without the addition of extra water. By leveraging the competitive solvation effect, this electrolyte effectively alleviates the kinetic sluggishness of Zn^2+^ transport inherent to binary eutectic systems, delivering deposition/stripping overpotentials comparable to those of aqueous electrolytes at 0.5 mA cm^−2^, while simultaneously improving zinc deposition morphology. Moreover, owing to competitive coordination, a greater fraction of anions participates in the Zn^2+^ solvation structure, which further induces the formation of a compact and robust SEI, thereby providing enhanced protection for the zinc anode. In addition, the water‐deficient nature of the ternary eutectic electrolyte effectively suppresses hydrogen evolution and zinc corrosion, resulting in an expanded electrochemical stability window of 2.53 V. The ternary eutectic electrolyte enhances the reaction kinetics in Zn||PANI cells, thereby improving both capacity and cycling stability. Moreover, benefiting from the excellent anti‐freezing capability of the eutectic electrolyte, the Zn||PANI cell retains 90.31% capacity after 1000 cycles at −20°C, while the printed pouch cell sustains stable operation for over 1300 h under the same conditions with a capacity retention of 98.46%. This work provides a viable and scalable pathway for the development of low‐cost, low‐polarization water‐deficient eutectic electrolytes for zinc‐based energy storage.

## Author Contributions

**Quancai Li**: conceptualization, methodology, software, investigation, formal analysis, Writing – original draft. **Jing Liang**: funding acquisition, visualization, writing – review and editing. **Jiayu Wang**: supervision, visualization, writing – review and editing. **Qian Wang**: software. **Hehe Ren**: visualization. **Ziyi Gong**: visualization. **Vellaisamy A. L. Roy**: investigation, visualization, writing – review and editing. **Wei Wu**: conceptualization, methodology, investigation, writing – review and editing, funding acquisition.

## Conflicts of Interest

The authors declare no conflicts of interest.

## Supporting information




**Supporting File**: advs77566‐sup‐0001‐SuppMat.docx.

## Data Availability

The data that supports the findings of this study are available in the supplementary material of this article.
