# Supplementary material for: Weakly Coordinated Solvent Network‐Induced Ternary Water‐Deficient Eutectic Electrolytes for Low‐Polarization Zn Anodes
Source: Adv Sci (Weinh). 2026 Sep 6:e77566. Online ahead of print. doi: 10.1002/advs.77566 (PMC13547542; doi:10.1002/advs.77566)
Supplement: Supplementary file 1 [file ADVS-9999-e77566-s001.docx]

**Supporting Information**

**Weakly Coordinated Solvent Network–Induced Ternary Water-Deficient Eutectic Electrolytes for Low-Polarization Zn Anodes**

Quancai Li#, Jing Liang#, Jiayu Wang#*, Qian Wang, Hehe Ren, Ziyi Gong, Vellaisamy A. L. Roy*, Wei Wu*

Quancai Li, Jing Liang, Qian Wang, Hehe Ren, Ziyi Gong, Wei Wu

*Laboratory of Printable Functional Materials and Printed Electronics, School of Physics and Technology, Renmin Hospital of Wuhan University, Wuhan University, Wuhan 430072, P. R. China*

Jing Liang, Vellaisamy A. L. Roy

*School of Science and Technology, Hong Kong Metropolitan University, Ho Man Tin, Hong Kong 999077, P. R. China*

Jiayu Wang

*Department of Clinical Laboratory, Institute of Translational Medicine, Renmin Hospital of Wuhan University, Jiefang Road 238, Wuhan 430060, P. R. China*

*Corresponding authors:

wangjiayu@whu.edu.cn (J. Wang)

[vroy@hkmu.edu.hk](mailto:vroy@hkmu.edu.hk) (VAL Roy)

[weiwu@whu.edu.cn](mailto:weiwu@whu.edu.cn) (W. Wu)

#Quancai Li, Jing Liang and Jiayu Wang contributed equally to this work.

**Experimental section**

**Materials**

Zinc sulfate heptahydrate (ZnSO_4_·7H_2_O, 99.5%), Ethylene glycol (EG, 99.5%), Methanol (MOH, 99.5), Aniline (99.5%), 1-Methyl-2-pyrrolidone (NMP, 99.0%) are purchased from Sinopharm Chemical Reagent Co., Ltd. Anhydrous zinc sulfate (ZnSO_4_) is purchased from Shanghai Macklin Biochemical Co., Ltd. Zinc powder is purchased from Aladdin reagent Co., Ltd. Acetylene black (Super P) is purchased from Taiyuan LZY Technology Co., Ltd.

**The preparation of electrolytes**

5.7512 g of ZnSO_4_·7H_2_O is stirred in deionized water to obtain a 2 M ZnSO_4_ aqueous solution, denoted as ZS. 5.7512 g of ZnSO_4_·7H_2_O is dissolved in EG or in a mixed solution of EG and MOH under ultrasonic treatment and stirring to obtain a 2 M eutectic electrolyte, denoted as ZE and ZEMX (X = 1, 3, 5, 7, for instance, X = 1 means MOH constitutes 10% of the total volume in the mixed solution of EG and MOH).

**The preparation of polyaniline (PANI) and electrode**

0.365 mL aniline monomer is added into 15 mL 1 M HCl. Subsequently, 0.228 g ammonium persulfate and 5 mL 1 M HCl are added into above solution under an ice bath, followed by continued stirring for another 1 h. Finally, dark green PANI is washed with deionized water and dried at 80 °C overnight. The PANI, super P, PVDF are fully ground with a mass ratio of 6:3:1 and dispersed in NMP solvent, then coated on Ti foil (diameter: 12 mm, thickness: 20 μm). The mass loading of PANI is 1-2 mg cm^-2^.

**The preparation of printable electrode inks**

The PANI, super P, and PVDF are fully ground at a mass ratio of 6:3:1, then added to NMP solution, and fully ultrasonic stirred to obtain PANI cathode ink. The zinc powder anode ink is obtained by fully dispersing zinc powder, super P, and PVDF in NMP solution at a ratio of 16:2:1.

**The preparation of printed Zn||PANI batteries (PZB)**

The printed Zn||PANI batteries are assembled via a sandwich structure. Commercial carbon paste is printed on polyethylene terephthalate as current collectors for the cathode and anode respectively, and dried at 120 °C for 30 min. Then, the prepared PANI ink and Zn powder ink are printed on the cathode and anode current collectors, respectively, and dried at 60 °C under vacuum for 12 h. Finally, the electrodes are assembled in a sandwich structure and packaged in a vacuum bag. All printing patterns are made using a 200 mesh screen. The mass loading of Zn powder is 8-10 mg cm^-2^, and the mass loading of PANI is 1-2 mg cm^-2^.

**Electrochemical measurements**

The performance tests of all cells (CR 2032) are conducted on a battery test system (LAND CT3002A) and an electrochemical workstation (CHI 760E). Zn||Zn symmetric cells are assembled by sandwiching the glass fiber (separators) between commercial Zn foil (diameter: 14 mm, thickness:50 μm). Zn||Cu and Zn||Ti cells are assembled using commercial Zn foil as anode, Cu/Ti foils (diameter: 14 mm, thickness: 20 μm) as cathode, and glass fiber (GF/A, Whatman) as separator (diameter: 18 mm). The amount of electrolyte is about 100 μL for per coin cell. The linear sweep voltammetry (LSV) measurement curves are carried out at a scan rate of 5 mV s^−1^. Chronoamperometry (CA) curves of Zn||Zn symmetrical cells are tested at -150 mV overpotential. The Tafel plot is measured at a rate of 1 mV s^-1^. The electrochemical impedance spectroscopy (EIS) is collected within 10^–2^-10^6^ Hz at an AC voltage of 5 mV in a symmetric Zn||Zn testing system. The coulombic efficiency (CE) test is measured with Zn||Cu asymmetrical cells with a Zn deposition of 1 mAh cm^−2^ at 1mA cm^−2^ and a charge cut-off voltage of 0.5 V.

**Characterizations**

Raman spectra is performed on XploRA Plus spectrometer and Horiba Lab RAM HR Evolution using a 532 nm laser, as for Raman spectra test, all the liquid electrolytes are sealed in capillary tubes. Attenuated total reflection-Fourier transform infrared (ATR-FTIR) is gathered by an IR spectrometer (IRTracer 100). The crystallographic information is collected by X-ray diffraction (XRD) with a SmartLab-3kW (Rigaku). DSC heating curves are collected using a differential scanning calorimeter (DSC3, Mettler-Toledo). The dynamic viscosity data of different electrolytes are obtained by a Kinexus pro+ rotational rheometer (Netzsch, Germany). X-ray photoelectron spectroscopy (XPS, ESCALAB Xi+, Thermo Fisher Scientific) is performed for analyzing the chemical composition. The morphology of materials is characterized with a cold field emission scanning electron microscope (SEM, Hitachi S-4800).

**Molecular dynamics (MD) simulations calculations**

MD simulations are performed by the Forcite module with COMPASS Ⅲ in Materials Studio to explore the solvation structure of Zn^2+^ ion in different electrolytes. Electrostatic interactions are treated using the Ewald sums, and Van der Waals (vdW) interactions were adopted using cut-off methods with a cut-off radius of 15.5 Å. After structure optimization, different ensemble processes are used to get a stable system under the conditions of 298 K for 500 ns respectively. After reaching the equilibrium states, radial distribution functions are calculated for the two systems above.

Figure S1 The cost corresponding to different mass specifications of zinc salts (Sigma-Aldrich).

Figure S2 (a) Optical photograph of ZE electrolytes with different salts (Left: ZE Ⅰ using ZnSO_4_·7H_2_O, right: ZE Ⅱ using ZnSO_4_). (b) Optical photograph of ZM (Solvent: MOH).

Taking the ZE electrolyte as an example (Figure S2a), when ZnSO_4_·7H_2_O is used as the zinc salt, the crystallization water acts as a Lewis acid, enabling complete dissolution in EG. In contrast, ZnSO_4_ cannot dissolve in EG, and a large amount of undissolved salt particles is observed.

ZnSO_4_·7H_2_O can also dissolve in MOH, with a solubility of less than 1 M. MOH not only acts as a hydrogen bond donor, but also serves as a diluent in the formation of ternary eutectic electrolytes.

Figure S3 EIS of different electrolytes using stainless||stainless cells and the corresponding conductivities.

Figure S4 The shear viscosity of different electrolytes at a shear rate of 100 s^-1^.

Figure S5 Calculation results of the total hydrogen bonds number between solvent molecules in different electrolytes.

Taking ZS, ZE, and ZEM5 as representative examples, molecular dynamics simulations are employed to quantify the total number of hydrogen bonds in the three systems. The results indicate that the hydrogen-bonding network of water molecules is substantially disrupted, thereby endowing the eutectic electrolytes with enhanced anti-freezing performance.

Figure S6 The contact angle test of different electrolytes.

Figure S7 RDF of Zn^2+^-O (H_2_O) in different electrolytes.

Figure S8 RDF of Zn^2+^-O (H_2_O) in different electrolytes.

Note: The chemical environment evolution of EG and MOH is investigated by analyzing the EG-CH_2_ and MOH-CH_3_ resonances. The EG-CH_2_ resonance located at 3.56 ppm in pristine EG shifts to around 3.47 ppm after introducing ZnSO_4_·7H_2_O to form the ZE electrolyte. This shift indicates that EG experiences a different chemical environment due to its interaction with Zn^2+^, suggesting that EG participates in the Zn^2+^ solvation structure rather than acting as a simple solvent. More importantly, the MOH-CH_3_ resonance located at 3.16 ppm exhibits a continuous downfield shift with increasing MOH content from ZE to ZEM1, ZEM3, and ZEM5. This systematic evolution demonstrates that the local chemical environment of MOH is progressively modified within the electrolyte network. If MOH merely acts as an inert diluent, the chemical environment of the methanol methyl group should remain largely unchanged with increasing MOH concentration. Therefore, the observed shift indicates that MOH participates in the reconstructed Zn^2+^ solvation network through interactions with Zn^2+^ and the surrounding hydrogen-bonding network.

Figure S9 Zn||Zn cells cycle performance of different electrolytes at 2 mA cm^-2^ and 4 mAh cm^-2^.

The Zn||Zn cell with ZS electrolyte shows the lowest overpotential but have a shot cycle life of 56 h. The ZE and ZEM1 electrolytes exhibited a significant increase in overpotential successively. ZEM3 experienced a short circuit after 240 h of cycling, while ZEM5 could undergo stable cycling for 330 h.

Figure S10 GITT profiles of different electrolyte systems.

Figure S11 Small angle X-ray scattering spectra of different electrolytes.

Figure S12 Differential capacitance plots of different electrolyte systems.

Figure S13 (a) EIS of hybrid capacitors and (b) The relative Relative diffusion coefficient of different electrolytes.

Figure S14 The function of MSD *vs.* time in ZS, ZE and ZEM5 electrolytes.

Figure S15 EIS of symmetrical cells for (a) ZS, (b) ZE, and (c) ZEM electrolytes at different temperatures.

Figure S16 DRT derived from *in situ* EIS during Zn plating and stripping of (a) ZE and (b) ZEM electrolyte systems after cycles. (c) Voltage-time curves.

The relaxation times (τ) of the four characteristic peaks in the DRT spectrum for the two electrolytes are nearly identical, yet they exhibit significant differences in impedance intensity at the same τ. This suggests that the ZE and ZEM dynamic paths are the same, but the resistances and contributions to polarization for each process differ substantially, which is clearly reflected in the polarization voltage.

Figure S17 CE of different electrolytes at 1 mA cm^-2^.

Figure S18 (a) SEM images of original zinc foil before soaking experiment. SEM images of zinc foil after soaking in (b) ZS electrolyte and (c) ZEM electrolyte systems for one week.

Figure S19 XRD patterns of Zinc foil immersed in different electrolytes for one week.

Figure S20 CV curve with ZS and ZEM5.

Figure S21 Chronoamperometry curves of ZS and ZEM5.

Figure S22 (a) XRD patterns of Zinc foils using ZEM5 with different cycles at 1 mA cm^-2^ and (b) the corresponding line chart for RTCs of 002, 100, and 101 Zinc planes, (c) Adsorption energy

Although solvent molecules exhibit stronger adsorption on the Zn(100) and Zn(101) planes than on the Zn(002) plane, this does not directly correspond to the preferred deposition plane. The stronger adsorption on Zn(100) and Zn(101) planes can effectively inhibit their growth kinetics, while the relatively weaker interaction on the Zn(002) plane facilitates Zn nucleation and deposition along this direction, resulting in the preferential Zn(002) texture.

Figure S23 (a) O 1s XPS spectra of the zinc anode with Ar^+^ sputtering. (b) XPS Auger spectra of Zn LMM regions. (c) TOF-SIMS of Zn after cycling with ZEM5.

Figure S24 Cycle performance of ZEM5 electrolyte at (a) 1.0 mA cm^-2^ and 1.0 mAh cm^-2^. (b) 1.0 mA cm^-2^ and 0.5 mAh cm^-2^.

Figure S25 Cycle performance of ZEM5 electrolyte at -20 °C.

Figure S26 (a) XRD pattern of PANI powder. (b) SEM image of PANI powder.

Figure S27 EIS of Zn||PANI cells with different electrolytes.

Figure S28 (a)The cycle performance of Zn||PANI with ZEM5 electrolyte at a current density of 0.5 A g^-1^.

Figure S29 (a)The cycle performance of Zn||PANI with ZEM5 electrolyte at -20 °C and the current density of 0.1 A g^-1^, (b) Voltage profiles of different cycles.

Figure S30 Thixotropic behavior of (a) PANI cathode ink and (c) Zn powder anode ink. Plots of viscosity versus shear rate for (b) PANI and (d) Zn power ink.

Note: Thixotropy refers to the decrease in ink viscosity under shear and its gradual recovery once the shear is removed, which is essential for printing stability and accurate pattern reproduction. Thixotropic tests simulating the squeegee action confirm that both the PANI cathode ink (a) and the Zn powder anode ink (c) possess suitable rheological properties for screen printing.

Rheological behavior is critical for reliable screen printing. As shown in (b) and (d), both inks exhibit typical shear-thinning behavior, ensuring stable ink transfer, precise pattern control, and minimized printing defects, thereby demonstrating their suitability for high-quality screen printing.

Figure S31 The optical image of the patterned electrode obtained through screen printing.

Figure S32 The thickness of micro-printed electrodes.

Figure S33 (a-c) SEM images of the printed PANI cathode and (d-f) zinc powder anode.

Figure S34 CV curve of printed Zn||PANI battery.

Figure S35 The rate performance of printed Zn||PANI battery.

Rate performance at room temperature: The printed Zn||PANI battery deliver specific capacities of 106.98, 93.63, 83.45, 75.46, 64.80, and 29.20 mAh g^-1^ at 0.1 to 2.0 A g^-1^.

Figure S36 (a)The cycle performance of printed Zn||PANI battery at a current density of 1.0 A g^-1^ and (b) Voltage profile.

Figure S37 The rate performance of printed Zn||PANI battery at -20 °C.

Rate performance at -20 °C: The printed Zn||PANI battery deliver specific capacities of 76.35, 72.67, 69.03, 66.33, and 63.88 mAh g^−1^ at 0.03 to 0.12 A g^−1^.

Figure S38 The cycle performance of printed Zn||PANI battery at -20 °C and a current density of 0.05 A g^-1^.

Figure S39 The cycle performance of printed Zn||PANI battery at -20 °C and a current density of 0.10 A g^-1^.

Figure S40 Optical image of printed cells connected in series lighting up a timer.

Table S1 The comparison of reported eutectic electrolytes

| Label | Material | Eutectic type | Literature |
| --- | --- | --- | --- |
| S1 | Zn(Ac)_2_ : Formamid = 1:9  Salt concentration : 2.8 M | Nonaqueous | Angew. Chem. Int. Ed. 2024, 136, e202406906 |
| S2 | ZnCl_2_ : Ethylene glycol = 1:4  Salt concentration : 4.45 M | Nonaqueous | Angew. Chem. Int. Ed. 2022, 134, e202206717 |
| S3 | Zn(BF_4_)_2_·xH_2_O : ZnCl_2_ : Sulfolane = 2:1:0.2  Salt concentration : — | Water-deficien | Angew. Chem. Int. Ed. 2025, 64, e202502761 |
| S4 | Choline chloride : Glucose : H_2_O = 2:1:10  Salt concentration of ZnSO_4_·7H_2_O : 2 M | Water-rich | Adv. Funct. Mater. 2025, 36, e15911 |
| S5 | Zn(OTf)_2_ : Urea : Dimethyl sulfoxide = 1:2:2 | Nonaqueous | Angew. Chem. Int. Ed. 2025, 61, e14392 |
| S6 | Choline chloride : N, N-dimethylformamide = 1:2  Salt concentration of ZnCl_2_ : 1 M | Nonaqueous | J. Am. Chem. Soc. 2025, 147, 39440 |
| S7 | Zn(BF_4_)_2_·xH_2_O : acetamide = 1:8  Salt concentration : — | Water-deficien | Adv. Energy Mater. 2024, 14, 2303549 |
| S8 | Levulinic acid : H_2_O = 1:1  Salt concentration of ZnSO_4_·7H_2_O : 2 M | Water-rich | Adv. Energy Mater. 2024, 15, 2403689 |
| S9 | Zn(ClO_4_)_2_·6H_2_O : Dimethyl sulfone : niacinamide = 1:3:0.5  Salt concentration : — | Water-deficien | Energy Environ. Sci. 2023, 16, 4502 |
| **This work** | ZnSO_4_·7H_2_O : Ethylene glycol : Methanol = 1:3.1:4.3  Salt concentration : 2 M | Water-deficient | — |
